# Rapid Isothermal DNA Amplification in Microchambers Detected by Fluorescence RNA Aptamer Transcription

**DOI:** 10.3390/diagnostics15222838

**Published:** 2025-11-09

**Authors:** Hideyuki Yaginuma, Ryoko Suzuki, Takako Akamatsu, Hiroyuki Noji, Kazuhito V. Tabata

**Affiliations:** 1 Department of Applied Chemistry, Graduate School of Engineering, The University of Tokyo, Tokyo 113-8656, Japan; 2 Sothis Technologies, Tokyo 113-0033, Japan

**Keywords:** isothermal DNA amplification, microchamber, digital assay, fluorescent aptamer, nucleic acid quantification

## Abstract

**Objectives:** Rapid detection and quantification of nucleic acids are essential for on-site diagnosis of pathogens. To provide an alternative to current methods that require bulky instruments and long reaction times, we developed a digital nucleic acid amplification method suitable for point-of-care applications. **Methods:** The method combines compartmentalization in micrometer-sized microchambers with recombinase polymerase amplification (RPA) and the Mango fluorescent aptamer system. Fluorescence microscopy was used to acquire images of microchambers. Single molecules of target DNA sequences were detected as fluorescence-positive chambers in the image and quantified by counting these chambers. **Results:** Detection and quantification were achieved within 8 and 22 min, respectively. The measurable concentration range was approximately 4 fM to 40 pM, demonstrating a wide dynamic range. Furthermore, the successful detection of five different pathogen-derived DNA sequences confirmed the versatility of the approach. **Conclusions:** Because the reaction proceeds isothermally within a compact microdevice, the system requires minimal instrumentation. These features make it a promising platform for nucleic acid measurement in point-of-care testing.

## 1. Introduction

Nucleic acid detection is essential for diagnosis and disease monitoring. In particular, the detection and quantification of pathogenic microorganisms, is important for containing infectious diseases [1]. In clinical circumstances, rapid tests based on antigen detection by lateral flow immunochromatography are commonly used. The assay reaction time of a typical rapid test is as short as 15 min. These tests do not provide quantitative results, and the sensitivity of immunochromatography is relatively low [2]. Therefore, nucleic acid tests are considered more effective for detecting very low levels of pathogens and reducing false negative results. Polymerase Chain Reaction (PCR) is considered one of the most sensitive methods for detecting pathogen-derived DNA and RNA. However, in many cases, PCR tests require samples to be sent to dedicated testing laboratories because thermal cyclers are expensive and sample handling requires trained professionals. In addition, the test reaction itself takes a long time, typically more than an hour. Because of these limitations, it takes days for test results to be returned to patients. Moreover, standard PCR tests do not provide quantitative results. Viral/bacterial load numbers can potentially be used to stratify the risks of patients and lead to more personalized therapeutics [3,4]. For these reasons, a rapid method to detect and quantify nucleic acids at the point of care is desired.

Recently, quantitative PCR (qPCR) has been widely used for nucleic acid quantification in both research and clinical settings [5]. By monitoring PCRs in real time, qPCR can report the amount of nucleic acids. The drawback is the relatively high cost of monitoring instrumentation, and the result is less reliable when PCR inhibitors are present in the reaction mix [6]. Alternatively, digital nucleic acid amplification tests (dNAAT) can be used to quantify nucleic acids. In dNAAT, the reaction fluid is separated into many small compartments so that each compartment contains either 0 or 1 target molecule. Under these conditions, the fraction of positive compartments can be converted to the absolute concentration of the analyte. As a result, the absolute concentration of nucleic acids can be quantified without the need for a calibration curve. Droplet digital PCR is one of these methods and shows improved quantification robustness compared to conventional qPCR [6,7,8,9,10]. The disadvantage of both qPCR and ddPCR is the bulky size and high cost of the instrument [11,12].

In theory, dNAAT testing can be performed at the point of care with simplified low-cost instruments, as long as the nucleic acid amplification reaction takes place in the small microcompartments and can be captured by a camera. The use of isothermal nucleic acid amplification reactions eliminates the need for thermal cycling. In addition, the microcompartments can be physically fabricated on a disposable chip, eliminating the need for a microfluidic droplet generation component in the instrument. Many nucleic acid detection reactions can be performed in such microchamber chips, including PCR [13,14], LAMP [15,16,17,18], RPA [19,20] and NASBA [21]. Currently, most of these methods suffer from relatively long assay times (typically 30–120 min) for the amplification reaction. These existing microcompartment systems use compartments of a size of tens to hundreds of microns or larger. To reduce the assay time, one approach may be to reduce the size of the microcompartments [22].

In this report, we present an alternative method to detect the target DNA in 8 min and measure it in 22 min by using single-digit µm-sized microcompartments. This method uses conventional RPA isothermal DNA amplification combined with the Mango fluorescent aptamer [23,24,25,26]. With this combination, we were able to develop one of the quickest digital isothermal NAAT-based detection and quantification of the target DNA molecule in the sample.

## 2. Materials and Methods

### 2.1. Microchamber Chip Preparation

Microchamber chips were fabricated as described previously [27]. Briefly, 24 × 32 coverslip (No. 1, Matsunami Glass, Kishiwada, Japan) was soaked in 0.05 vol % (3-aminopropyl)triethoxysilane (TCI, Tokyo, Japan), spin-coated with CYTOP (CTL-816AP, AGC Chemicals, Tokyo, Japan) and then coated with positive photoresist AZ P4903 (Merck, Darmstadt, Germany). The photoresist was UV-irradiated through a chrome-coated photomask patterned with 3 µm diameter pores and a pitch of 9 µm pitch using a mask aligner (BA100it, Nanometric Technology Inc., Tokyo, Japan). The photoresist was developed using AZ 300 MIF developer (Merck) and then the CYTOP layer was etched with O_2_ plasma by reactive ion etching (RIE-10NR, Samco, Kyoto, Japan). The photoresist was removed with acetone, and the coverslip was rinsed with 2-propanol and deionized water. Each microchamber had a diameter of 4.0–4.4 µm and a depth of 3.0–3.3 µm. The diameter and depth of the microchambers were measured using VK-X200 (Keyence, Osaka, Japan).

Flow cells were prepared using double-sided tape and a design seal cutter. A single chip had 4 flow cells (Supplementary Figure S1). The assay chip was assembled prior to reaction mix preparation.

### 2.2. T7 Mango RPA Reaction

Reaction premix without template nucleic acids (2.1 µL each of 10 µM forward and reverse primers, 1 TwistAmp Basic pellet (Abbott, Abbott Park, IL, USA), 29.5 µL of rehydration buffer of TwistAmp Basic, 1 µL of 50 µM TO-1 Biotin (ABM, Vancouver, BC, Canada), 0.75 µL of 1,000,000 U/mL TT7 polymerase (TOYOBO, Osaka, Japan), 2 µL of 25 mM NTP (Thermo Fisher Scientific, Waltham, MA, USA), 0.5 µL of 1% [*v*/*v*] S-386 (AGC Seimi Chemicals, Chigasaki, Japan), 1.25 µL of 200 µM Alexa Fluor 647 (Thermo Fisher Scientific), 5.8 µL of RNase-free water, and 2.5 µL of template DNA in total 47.5 µL reaction premix) was prepared at RT. Then, to start the amplification reaction, 2.5 µL of 280 mM magnesium acetate (Abbott, USA) was added to make a total of 50 µL of reaction mix. This reaction mix was introduced into the microfluidic flow channel of the microchamber chip. The chip was sonicated for 1 s to remove air bubbles in the microchambers. The chip was then incubated at RT for 100 s unless otherwise stated, and microchamber sealing oil (AE3000 (AGC Chemicals, Japan), 1% S-386) was introduced into the flow channel. For the endpoint assays, the chip was placed on a heat block set at 39 °C for 30 min, placed on a microscope and images were acquired. For the time-lapse measurements, the chip was placed on a temperature-controlled microscope stage and images were acquired sequentially. The stage was set 2 degrees higher than the desired temperature so that the microchamber side of the flow cell was at the desired temperature.

The oligonucleotide sequences of the RPA primers and templates are summarized in Supplementary Table S1.

### 2.3. Imaging

Images were captured using an Olympus IX83 microscope equipped with a 20× objective lens, LED light source (Xcite XYLIS II, Excelitas, Pittsburgh, PA, USA), and sCMOS camera (Andor Neo, Oxford Instruments, Oxford, UK). Bright field, Cy5 and GFP channel images were acquired sequentially. The Cy5 channel was used to image AF647, and the GFP channel was used to image the Mango aptamer.

### 2.4. Semi-Automated Image Analysis

Images were analyzed semi-automatically with a home-made macro of ImageJ/Fiji (version 1.54m) [28] in a manner similar to previously shown methods [27]. Briefly, each microchamber was detected based on the AF647 fluorescence channel image. Under our imaging conditions, each microchamber appears as a circle of ~10 pixels (px) in diameter. To measure the fluorescence intensity, a circular region of interest (ROI) of 10 px in diameter was created for each microchamber, and the average intensity of Mango aptamer fluorescence was calculated. For the calculation of the radius of gyration (ROG), a 16 px × 16 px square region was cropped around each microchamber. Then, the following formula was used to calculate the ROG of the cropped image,

$$ROG=\sqrt{\frac{m_{20}+m_{02}}{m_{00}}}$$

where

$$m_{20}=\sum_{y=0}^{M-1}\sum_{x=0}^{N-1}{\left\{x-\frac{\left(N-1\right)}{2}\right\}}^{2}g\left(x,y\right)$$

$$m_{02}=\sum_{y=0}^{M-1}\sum_{x=0}^{N-1}{\left\{y-\left(M-1\right)/2\right\}}^{2}g\left(x,y\right)$$

$$m_{00}=\sum_{y=0}^{M-1}\sum_{x=0}^{N-1}g\left(x,y\right)$$

M and N are the height and width of the image in pixels, and $g\left(x,y\right)$ is the signal intensity at pixel position $\left(x,y\right)$. The position coordinates, intensity of Mango fluorescence and ROG for all the ROIs in an image were calculated and exported as text files. These text files were then imported into a home-made Python 3.7 code for statistical analysis and plotting. The code used for statistical analysis is uploaded to GitHub (https://github.com/yag0123/Isothermal-DNA-Amplification-in-Microchambers, accessed on 30 April 2025).

For some negative control samples in Figure 4, some microchambers with autofluorescence from air bubbles or aggregates of unknown identity were occasionally classified as fluorescence positive. Because these false-positive microchambers in the image can be discriminated by eye from reaction-positive microchambers, we omitted these data points from analysis. The data before and after this manual modification is shown in Supplementary Table S2.

Mean + 15 SD threshold values were determined using negative control samples, except for time-lapse assays, where we used values from the first frame. SD was determined by fitting the intensity histogram to a Gaussian distribution.

### 2.5. Calculation of Estimated Template Concentration

The estimated concentration of template oligonucleotides ($c_{E}$) in our assay was calculated as follows,

$$c_{E}=\lambda_{exp}/\left(V\cdot N_{A}\right)$$

where $\lambda_{exp}$ is the fraction of reaction-positive microchambers obtained by experiment, $N_{A}$ is the Avogadro’s number and V is the average volume of the microchambers. V is calculated using the following equation,

$$V={\left(d/2\right)}^{2}\cdot \pi \cdot h$$

where d and h are the average diameter and depth of the microchambers, respectively.

## 3. Results

### 3.1. T7-Mango DNA Detection in Microchambers

**Figure 1:**
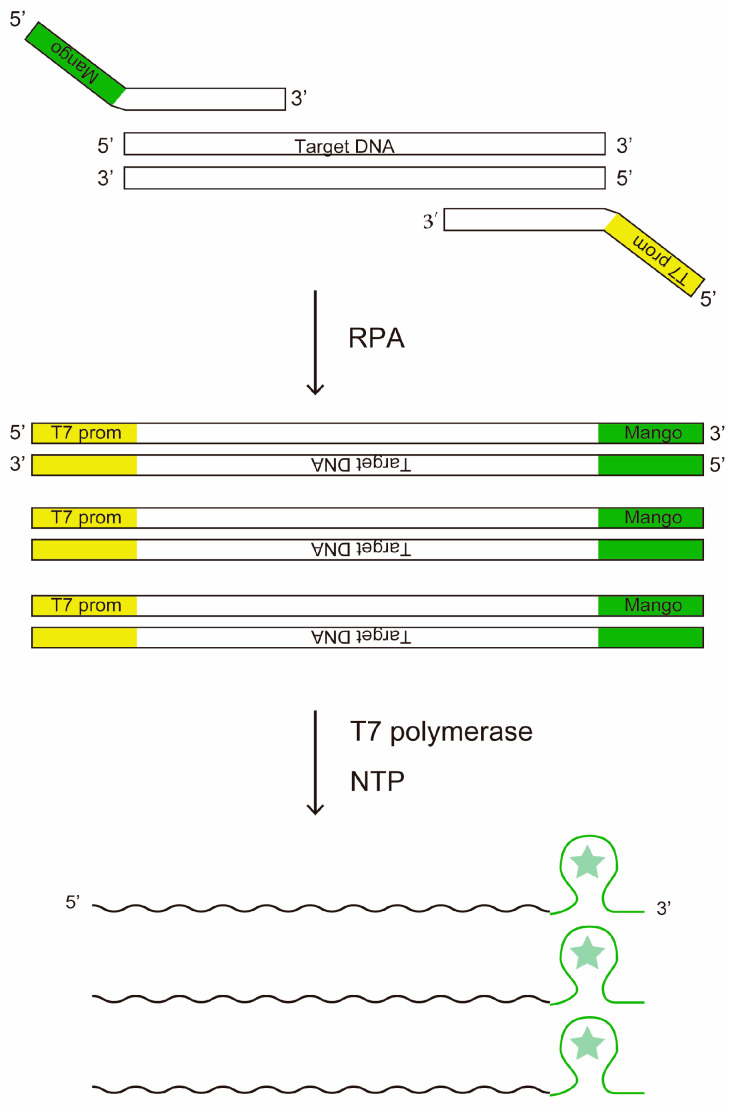
The principle of DNA amplification and detection by RPA combined with T7 transcription. The star symbol stands for the fluorophore of the fluorescent aptamer.

We conceived of combining RPA with the Mango aptamer for amplification of single molecule nucleic acids trapped in µm-sized microchambers. Instead of thermal cycling in PCR, RPA uses T4 phage-derived recombinases to load primers onto double-stranded template DNA to achieve isothermal DNA amplification at ~40 °C. Upon successful primer loading, Bsu DNA polymerase in the reaction mix catalyzes both the removal of the existing reverse-complement strand and the synthesis of reverse-complement DNA [29]. Mango aptamer is a fluorescent RNA aptamer that emits green fluorescence when bound to its substrate compound TO1-biotin [23,24]. In our hypothesis, by attaching Mango and T7 promoter to the forward and reverse primers of RPA, we would be able to detect amplification of target DNA in microchambers by Mango fluorescence (Figure 1).

**Figure 2:**
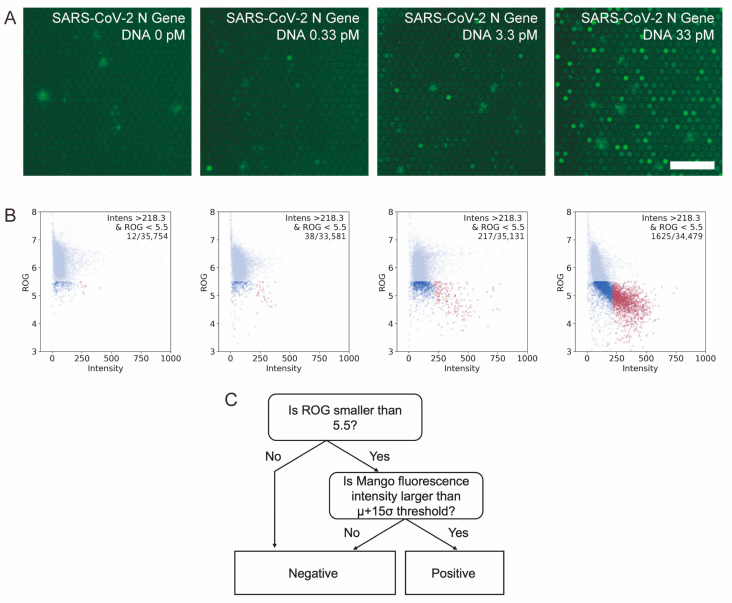
Fluorescence signal from the Mango aptamer after the DNA amplification reaction. (**A**) Fluorescence images of microchambers after reaction at different template concentrations. Bar = 50 µm. (**B**) 2D plot of fluorescence intensity vs. ROG for each template concentration. Red spots show the positive microchambers judged by our detection criteria. Light blue and blue spots show the negative microchambers. (**C**) Logic tree for judging positive microchambers.

To test this idea, we designed a model system that amplifies a DNA fragment with a sequence derived from the SARS-CoV-2 N protein in microchambers. In addition to standard RPA components, T7 polymerase, TO1-biotin and NTP were included in the reaction mix. By optimizing the concentration of NTP included in the system in the preliminary experiments, we found that an NTP concentration of 1 mM gave the largest number of fluorescence-positive chambers. The reaction mixture was prepared and introduced into the microfluidic flow channel created above the microchambers. After a short incubation at RT, water-immiscible fluorinated oil was introduced into the channel to separate the microchambers [30]. After 30 min incubation at 39 °C, we found that fluorescence increased in a fraction of the microchambers, and higher initial target DNA concentrations appeared to have more fluorescence-positive microchambers (Figure 2A).

Since there are thousands of microchambers in a single image, we used semi-automated image analysis for rapid quantification of fluorescence-positive microchambers. Using a home-made image analysis macro, we detected the positions of each microchamber and measured the fluorescence intensity for each of them. In addition, to reduce the chance of counting false-positive chambers containing impurities with strong autofluorescence, we calculated the ROG value of a single microchamber image for each microchamber [27]. If the microchamber is fluorescence-positive, the 16 × 16 pixel cropped image of a single microchamber will have bright pixels in the central area, and the ROG value will be small. In contrast, if the microchamber is fluorescence-negative or a large fluorescent object exists in the cropped image, the image would have a similar intensity throughout the image, and the ROG value will be large. Therefore, in our analysis, a microchamber is classified as negative if the ROG value is greater than the threshold value, and then the remaining microchambers were classified as negative or positive depending on their fluorescence intensity (Figure 2B). Through such automated image analysis, we confirmed that the number of fluorescence-positive microchambers indeed increased with increasing the initial amount of target DNA. The results suggest that RPA combined with Mango can successfully detect and quantify the amplification of nucleic acids in microchambers. When the RT incubation time prior to fluorinated oil separation was extended, we observed an increase in false positive chambers (Supplementary Figure S2). To facilitate sample preparation, the RT incubation time was set to 100 s in the following experiments.

### 3.2. Quantification of DNA Enabled in 22 min

**Figure 3:**
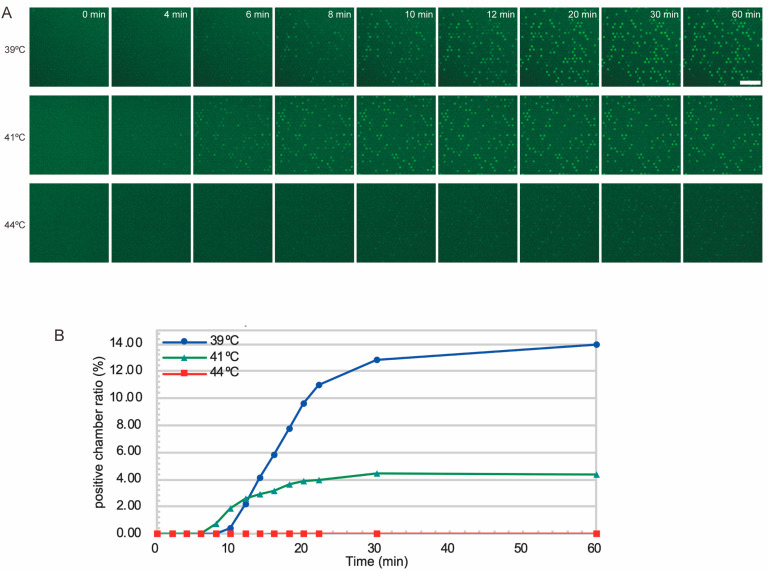
Timelapse imaging of microchambers during T7 Mango DNA amplification reaction at different temperatures. (**A**) Sequential fluorescence images of microchambers. Bar = 50 µm. (**B**) Fraction of positive microchambers plotted over time.

To investigate the time required for signal detection, we observed the increase in fluorescence-positive microchambers by time-lapse imaging. In this study, the detection time and quantification time were defined as follows. Detection time was determined as the time when a clear increase in the number of fluorescence-positive microchambers was observed from the initial state. Specifically, it was defined as the time when the number of positives per image, initially ≤2, exceeded 10 (corresponding to approximately 0.1% of the total microchambers). Quantification time was defined as the time when the change in positives became sufficiently small. More specifically, when images were acquired every two minutes, it was the first time after the detection time that the change in signal between consecutive images fell below 20% of the previous image. Based on these definitions, the detection time in our measurements was 8 min, and the quantification time was 22 min (Figure 3A,B and Figure S3). The concentration estimated from the positive microchamber fraction after 30 min was 5.0 pM, which was in good agreement with the theoretical concentration (3.6 pM). We also tested whether increasing the incubation temperature would result in earlier detection of positive chambers. At 41 °C, the final fraction of positive chambers was lower than at 39 °C, suggesting a lower signal-to-noise ratio. At 44 °C, we found very few positive microchambers. These results suggest that at higher temperatures, the activity of the enzymes decreases and gives compromised results. Therefore, we chose 39 °C as the incubation temperature for this system in the later experiments.

### 3.3. Successful Quantification of Multiple Nucleic Acid Sequences

**Figure 4:**
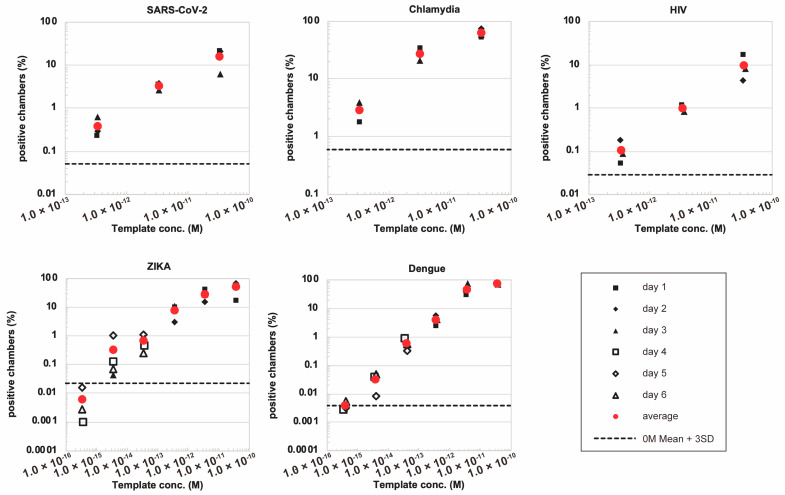
Detection of various nucleic acid sequences derived from different infectious microorganisms. The fraction of positive microchambers was measured at different template concentrations. The horizontal dotted line represents the mean + 3 SD level of the negative control.

Next, in order to verify the versatility of this system, we performed the detection of a variety of nucleic acid sequences other than SARS-CoV-2. We synthesized DNA sequences derived from HIV, Chlamydia, Zika and Dengue, as well as corresponding oligonucleotide primers with Mango aptamer and T7 promoter sequences. To evaluate the reproducibility of the experiment, we performed the assay 3–6 times on different days for each template. We found that the number of positive chambers after incubation correlated linearly with the concentration of input DNA fragments in these pathogen-derived DNA amplification models (Figure 4 and Supplementary Table S2). The measurements performed on different days were consistent, showing good reproducibility of the assay. This suggests that DNA quantification by RPA combined with the Mango aptamer in microchambers can be applied to different types of DNA sequences. The estimated concentration using our new method was generally consistent with the theoretical concentration, although a deviation of approximately one order of magnitude, either up or down, was observed depending on the target. Overall, our data suggest that microchamber-based digital RPA combined with the Mango aptamer has the potential to measure DNA concentration.

## 4. Discussion

Here, we show that detection of DNA amplification from single-molecule templates in microchambers can be achieved by combining the T7 promoter, the Mango fluorescent aptamer and RPA. We showed that the number of fluorescence-positive chambers after incubation at 39 °C correlated linearly with the initial DNA concentration. This suggests that this Mango-RPA reaction can be used to measure the concentration of DNA molecules in the sample. The detection time was 8 min and the measurement time was 22 min at 39 °C without thermal cycling, suggesting that this reaction can be completed rapidly by simply placing the reaction chip in a temperature-controlled environment.

The number of positive chambers can be used to estimate the initial concentration of target DNA using the average volume of the microchambers. The estimated template concentration was close to the theoretical concentrations, although different primer and template combinations gave slightly different results. Since the primers used in this work were not optimized, this variation could be explained by the different RPA and T7 transcription efficiencies depending on the primer and/or template sequence. For practical use as a diagnostic kit, it would be advisable to determine in advance the expected positive chamber rate for each primer and make this information available to end users. In our results, the coefficient of variance percentage (CV%) of positive microchamber counts was 8.7–150%, with a median value of 53%. In the case of qPCR, the CV of estimated copy numbers was less than 10% under ideal reaction conditions, while it was as high as 13–86% when contaminants were present in the reaction mix [10]. Therefore, the values measured by the T7-Mango RPA method have a similar variance to qPCR in sub-optimal conditions. The considerable variability observed in this study is likely due to the instability of nucleic acid amplification reactions initiated from single molecules within individual microchambers. In our assay, uneven reaction efficiencies were occasionally observed across different positions on the microchamber chip. Such variation may stem from inconsistencies in chip quality during in-lab fabrication or from positional differences in sealing conditions with fluorinated oil, including the oil flow rate. Future optimization of fabrication and sealing parameters is expected to improve the stability and reproducibility of the assay.

The lowest limit of detection (LoD) of the DNA detection method presented here was between 1 fM and 100 fM, depending on the target. 1 fM corresponds to approximately 2000 copies of DNA in 1 µL of sample. Rapid tests based on immunochromatography can detect down to 10^4^ copies per µL [31]. PCR tests typically detect very low copy numbers (<100) per reaction. Thus, in terms of sensitivity, our new method is currently similar to or slightly better than the immunochromatography test, but may not be as good as PCR. In future work, it is desirable to achieve a lower detection limit. One way to achieve the improvement of LoD in our assay may be to capture and concentrate the target DNA molecule on the solid phase instead of simple diffusion [32,33].The reaction presented here can be used to develop a quantitative pathogen dNAAT test that can be performed at the point-of-care (POC) without large instrumentation. In our study, we selected synthesized DNA with the sequence of pathogen nucleic acids as a model system to demonstrate our assay. For the detection of RNA viruses using the method shown in this study in a real-world setup, further development of steps for RNA extraction and reverse transcription will be required at the POC. In addition, it is desirable to develop a portable imaging device with temperature control that can be used at POC in the future. In our imaging approach, it is sufficient to count the number of chambers exhibiting fluorescence signals; therefore, a high-resolution camera or microscope is not necessarily required. Provided that an appropriate optical system is designed, a general-purpose imaging device such as a smartphone camera could potentially provide quantification performance comparable to that of a research-grade fluorescence microscope. In combination with a smartphone-based imaging device such as that presented in our previous work [34], it may be possible to measure the DNA using a smartphone camera.

## 5. Conclusions

T7 and Mango combined with RPA can be used to detect and quantify target DNA quickly. This reaction can potentially be used to create a low-cost and portable POCT for pathogens in the future.

## Data Availability

The raw data of this study and the codes used for analysis were uploaded to zenodo.org (https://doi.org/10.5281/zenodo.15287439, accessed on 7 November 2025).

## Supplementary Materials

The following supporting information can be downloaded at: https://www.mdpi.com/article/10.3390/diagnostics15222838/s1, Figure S1: In-house fabricated microchamber chip used in this study; Figure S2: Fraction of positive microchambers when RT incubation time before starting the reaction at 39 °C was varied; Figure S3: Histogram of fluorescence intensity during timelapse measurement; Table S1: Oligonucleotide sequences used in this study; Table S2: Number of positive counts in negative control experiments. References [35,36,37,38] were cited in Supplementary Materials.
