# Supplementary material for: Rapid Isothermal DNA Amplification in Microchambers Detected by Fluorescence RNA Aptamer Transcription
Source: Diagnostics (Basel). 2025 Nov 9;15(22):2838. doi: 10.3390/diagnostics15222838 (PMC12650952; doi:10.3390/diagnostics15222838)
Supplement: Supplementary file 1 [file diagnostics-15-02838-s001.zip › 20250927SupplementaryInformation.pdf]

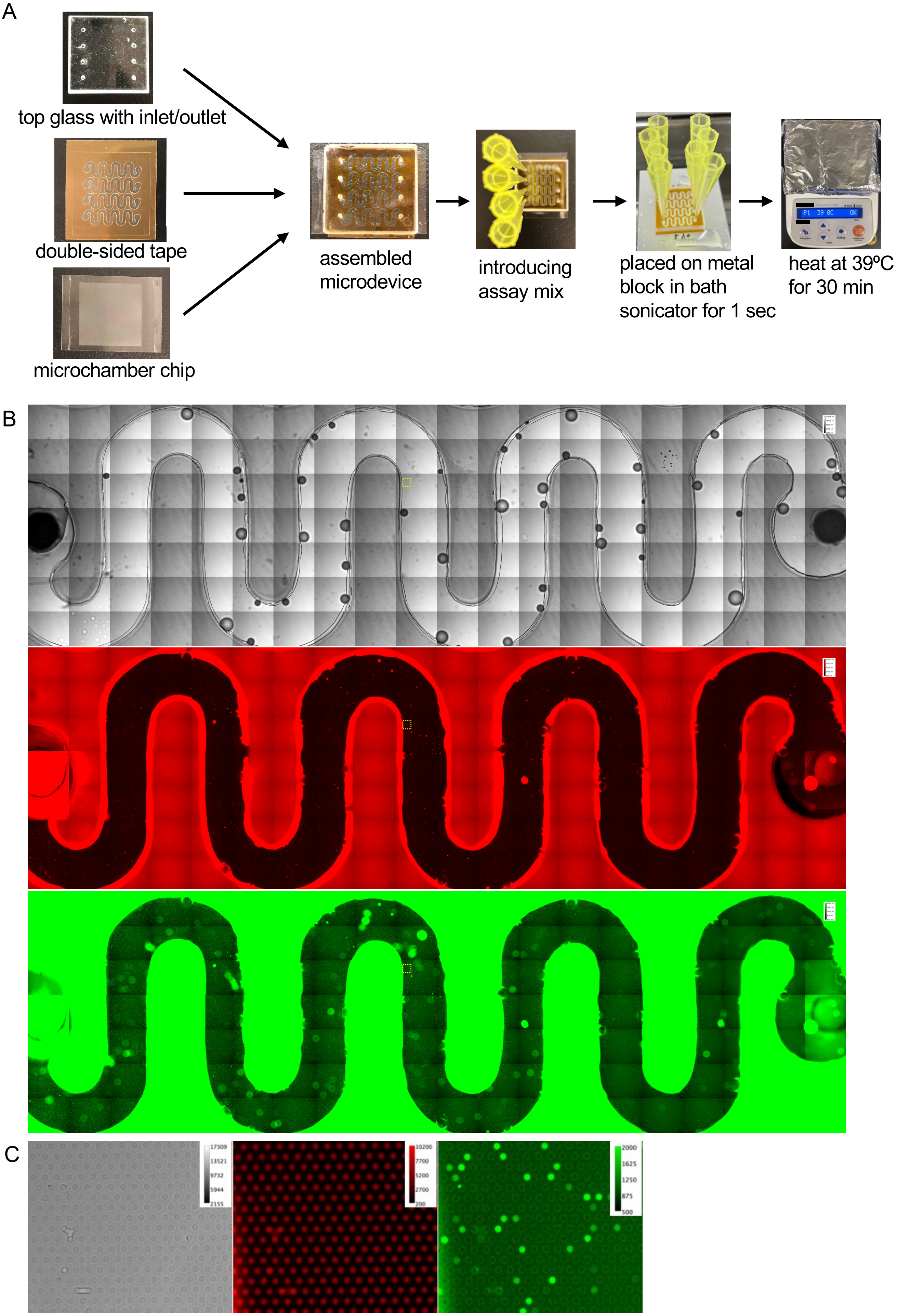

Supplementary Fig. S1

In-house fabricated microchamber chip used in this study. (A) Overview of chip assembly and digital assay procedure. (B) Multi-image array of the flow cell of the microchamber chip. Bright field (Gray), Cy5 setting (Red, for Alexa Fluor 647 fluorescence) and GFP setting (Green, for Mango fluorescence) of the microscope is shown. (C) Magnified image of the area of yellow rectangle in (B).

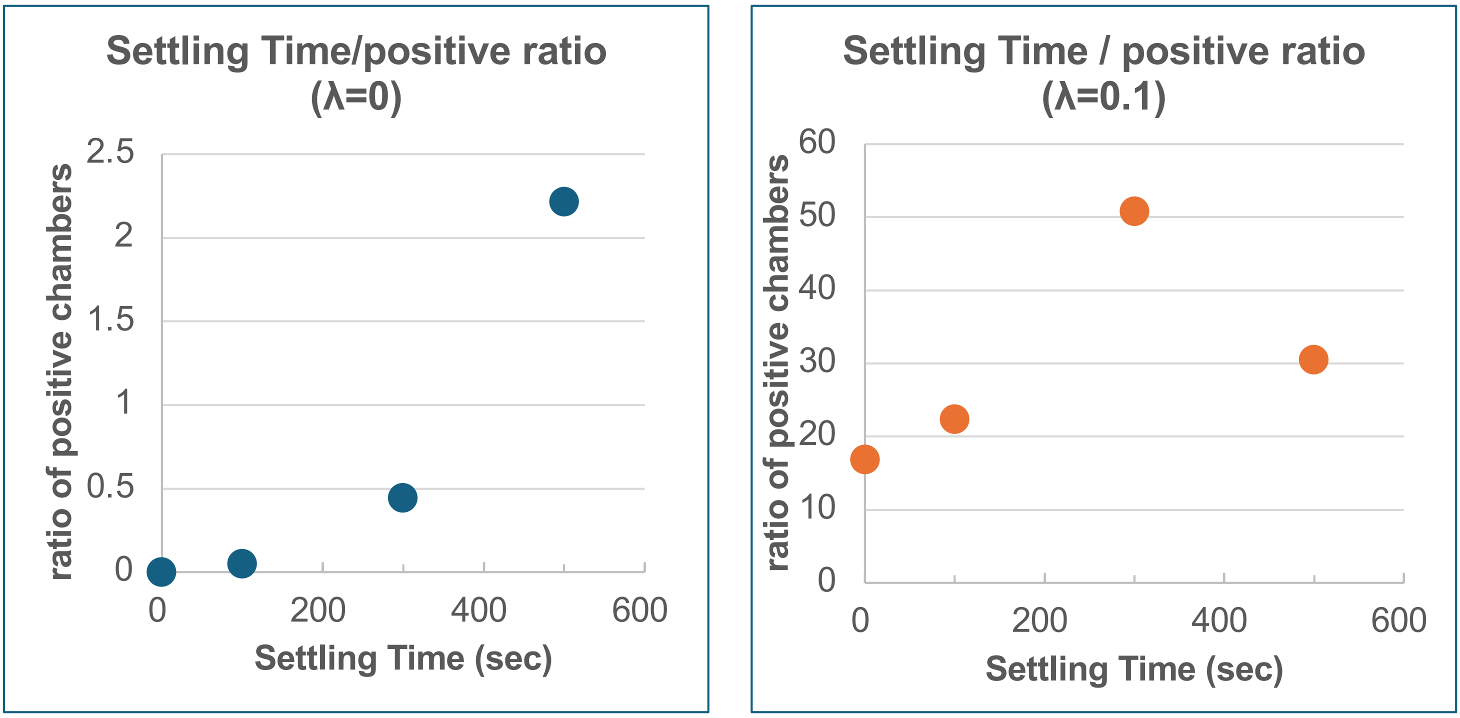

Supplementary Fig. S2
Fraction of positive microchambers when RT incubation time before starting the reaction at 39ºC was varied. Left: no template control, right: 4 pM template condition.

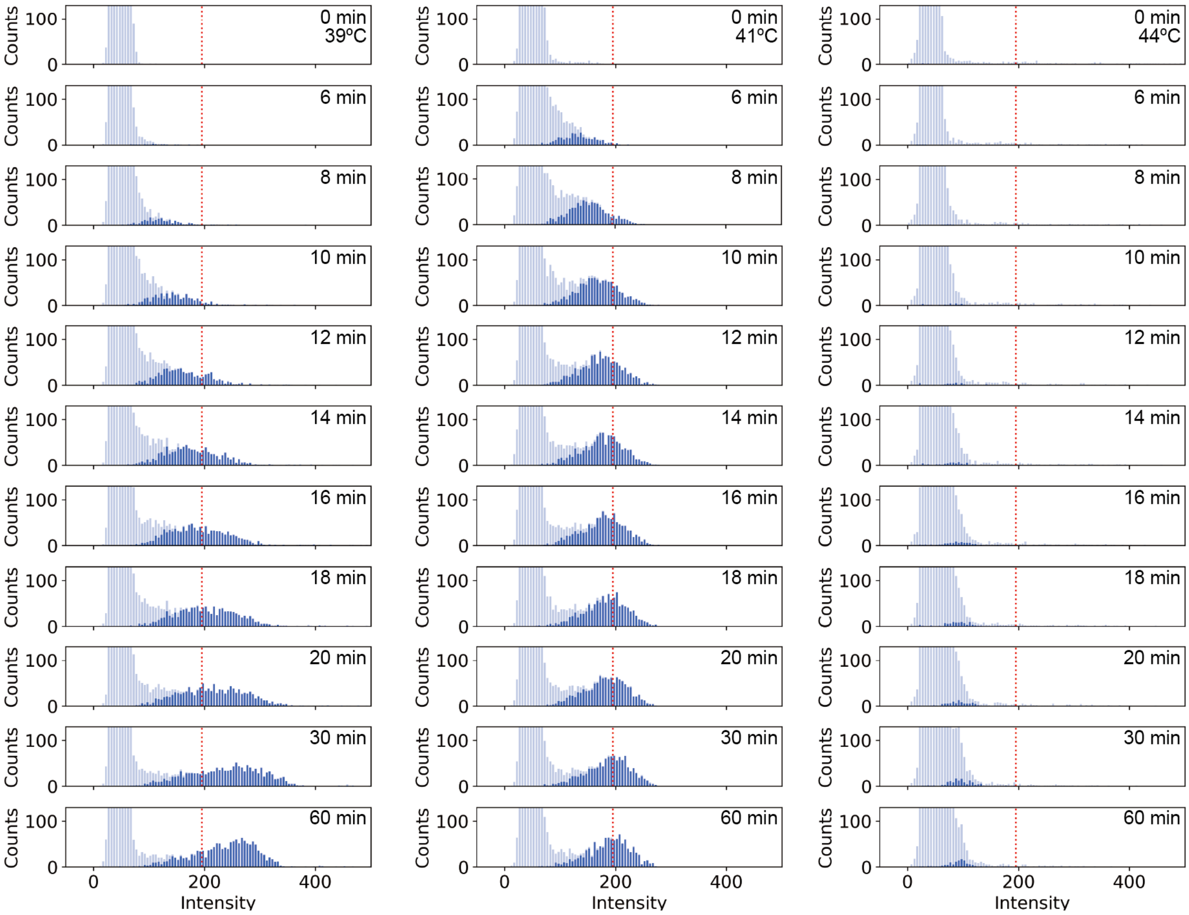

Supplementary Fig. S3

Histogram of fluorescence intensity during timelapse measurement. Vertical red dotted line indicates the mean + 15SD threshold signal level at 0 s. Related to Fig. 3.

Name	Sequence†	Base pairs	Reference
SARS-Cov-2 N Gene Template	ctgagggagccttgaatacaccaaaagatcacattggcacccgcaatcctgctaacaatgctgc aatcgtgctacaacttcctcaaggaacaacattgccaaaaggcttctacgcagaagggagcag aggcggcagtcaagcctcttctcgttcctcatcacgtagtcgcaacagttcaag	181	GenBank OU610953.1
SARS-Cov-2 Mango-Fwd Primer	GGCACGTACGAATATACCACATACCAAACCTTCCTTCGTACGTGCCctgagg gagccttgaatacaccaaaagatcac	78	This work
SARS-Cov-2 T7-Rev Primer	GAATTTAATACGACTCACTATAGGGAGACCcttgaactgttgcgactacgtgatga ggaacgag	64	This work
Chlamydia CDS2 Template	caaatatcatctttgcggttgcgtgtcctgtgaccttcattatgtcggagtctgagcaccctaggcg tttgtactccgtcacagcggttgctcgaagcacgtgcggggttattttaaaagggattgcagcttg tagtcctgcttgagagaacgtgcgggcgatttgccttaa	172	NCBI Reference Sequence NC_001372.1
Chlamydia Mango-Fwd Primer	GGCACGTACGAATATACCACATACCAAACCTTCCTTCGTACGTGCCttaagg caaatcgcccgcacgttctctcaagc	78	Ref. S1[1]
Chlamydia T7-Rev Primer	GAATTTAATACGACTCACTATAGGGAGACCcaaatatcatctttgcggttgcgtgtc ctgtg	62	Ref. S1[1]
HIV pol Template	tggcagtattcattcacaattttaaaagaaaaggggggattggggggtacagtgcaggggaaa gaatagtagacataatagcaacagacatacaaactaaagaattacaaaaacaaattacaaaa attcaaaattttcggg	141	GenBank AF033819.3
HIV Mango-Fwd Primer	GGCACGTACGAATATACCACATACCAAACCTTCCTTCGTACGTGCCtggcag tattcattcacaattttaaaagaaaagg	80	Ref.S2[2]
HIV T7-Rev Primer	GAATTTAATACGACTCACTATAGGGAGACCcccgaaaattttgaatttttgtaattt gtttttg	64	Ref.S2[2]
Dengue Template	aacagcatattgacgctgggagagaccagagatcctgctgtctcctcagcatcattccaggcac agaacgccagaaaatggaatggtgctgttgaatcaacaggttc	107	GenBank AY662691.1
Dengue Mango-Fwd Primer	GGCACGTACGAATATACCACATACCAAACCTTCCTTCGTACGTGCCcagcat attgacgctgggagagaccagagatcc	79	Ref. S3[3]
Dengue T7-Rev Primer	GAATTTAATACGACTCACTATAGGGAGACCgaacctgttgattcaacagcaccatt ccatttt	63	Ref. S3[3]
Zika Template	tctcttggagtgcttgtgattctactcatggtgcaggaggggttgaagaagagaatgaccacaaa gatcatcatgagcacatcaatggcagtgctggtagtcatgatcttgggaggattttcaatgagtg acctggccaagc	142	GenBank LC002520.1
Zika Mango-Fwd Primer	GGCACGTACGAATATACCACATACCAAACCTTCCTTCGTACGTGCCtctcttg gagtgcttgtgattctactcatggt	78	Ref. S4[4]
Zika T7-Rev Primer	GAATTTAATACGACTCACTATAGGGAGACCgcttggccaggtcactcattgaaaat cctc	60	Ref. S4[4]

Supplementary Table S1. Oligonucleotide sequences used in this study.

†Mango and T7 sequences are highlighed in capital letters.

	Number of microchambers		
Experiment	Positives	Total	% of positives
SARS-CoV-2 day 1 negative control	9(12)	34927	0.026(0.034)
SARS-CoV-2 day 2 negative control	0(7)	35560	0.000(0.020)
SARS-CoV-2 day 3 negative control	1(4)	36790	0.003(0.011)
Chlamydia day 1 negative control	1(129)	34578	0.003(0.373)
Chlamydia day 2 negative control	0(22)	35991	0.000(0.061)
Chlamydia day 3 negative control	97(176)	33823	0.287(0.520)
HIV day 1 negative control	0(4)	31080	0.003(0.013)
HIV day 2 negative control	2(5)	33756	0.006(0.015)
HIV day 3 negative control	5(19)	35203	0.014(0.054)
Dengue day 1 negative control	1(9)	35443	0.003(0.025)
Dengue day 2 negative control	0(1)	33866	0.000(0.003)
Dengue day 3 negative control	0(13)	33482	0.000(0.039)
Dengue day 4 negative control	0(10)	33064	0.000(0.030)
Dengue day 5 negative control	0(2)	32141	0.000(0.006)
Dengue day 6 negative control	0(5)	36715	0.000(0.014)
Zika day 1 negative control	0(3)	38537	0.000(0.008)
Zika day 2 negative control	0(0)	31197	0.000(0.000)
Zika day 3 negative control	0(198)	29667	0.000(0.667)
Zika day 4 negative control	6(9)	38004	0.016(0.024)
Zika day 5 negative control	0(5)	34961	0.000(0.014)
Zika day 6 negative control	1(8)	35381	0.003(0.023)

Supplementary Table S2. Number of positive counts in negative control experiments.

Numbers before omitting the obvious false positives discriminated by eye are shown in parentheses.

Supplementary References

S1. J. Zhai, L. Wang, X. Qiao, J. Zhao, X. Wang and X. He, *PloS one*, 2021, **16**, e0251119.

S2. D. S. Boyle, D. A. Lehman, L. Lillis, D. Peterson, M. Singhal, N. Armes, M. Parker, O. Piepenburg and J. Overbaugh, *Mbio*, 2013, **4**.

S3. B. T. Teoh, S. S. Sam, K. K. Tan, M. B. Danlami, M. H. Shu, J. Johari, P. S. Hooi, D. Brooks, O. Piepenburg, O. Nentwich, A. Wilder-Smith, L. Franco, A. Tenorio and S. AbuBakar, *Journal of clinical microbiology*, 2015, **53**, 830-837.

S4. A. Abd El Wahed, S. S. Sanabani, O. Faye, R. Pessoa, J. V. Patriota, R. R. Giorgi, P. Patel, S. Bohlken-Fascher, O. Landt, M. Niedrig, P. M. Zanotto, C. P. Czerny, A. A. Sall and M. Weidmann, *PLoS Curr*, 2017, **9**.
